# Artificial Intelligence in Biological Sciences

**DOI:** 10.3390/life12091430

**Published:** 2022-09-14

**Authors:** Abhaya Bhardwaj, Shristi Kishore, Dhananjay K. Pandey

**Affiliations:** Amity Institute of Biotechnology, Amity University Jharkhand, Ranchi 834001, India

**Keywords:** artificial intelligence, biotechnology, agriculture, medicine, crop yield, life science

## Abstract

Artificial intelligence (AI), currently a cutting-edge concept, has the potential to improve the quality of life of human beings. The fields of AI and biological research are becoming more intertwined, and methods for extracting and applying the information stored in live organisms are constantly being refined. As the field of AI matures with more trained algorithms, the potential of its application in epidemiology, the study of host–pathogen interactions and drug designing widens. AI is now being applied in several fields of drug discovery, customized medicine, gene editing, radiography, image processing and medication management. More precise diagnosis and cost-effective treatment will be possible in the near future due to the application of AI-based technologies. In the field of agriculture, farmers have reduced waste, increased output and decreased the amount of time it takes to bring their goods to market due to the application of advanced AI-based approaches. Moreover, with the use of AI through machine learning (ML) and deep-learning-based smart programs, one can modify the metabolic pathways of living systems to obtain the best possible outputs with the minimal inputs. Such efforts can improve the industrial strains of microbial species to maximize the yield in the bio-based industrial setup. This article summarizes the potentials of AI and their application to several fields of biology, such as medicine, agriculture, and bio-based industry.

## 1. Introduction

**Figure 1:**
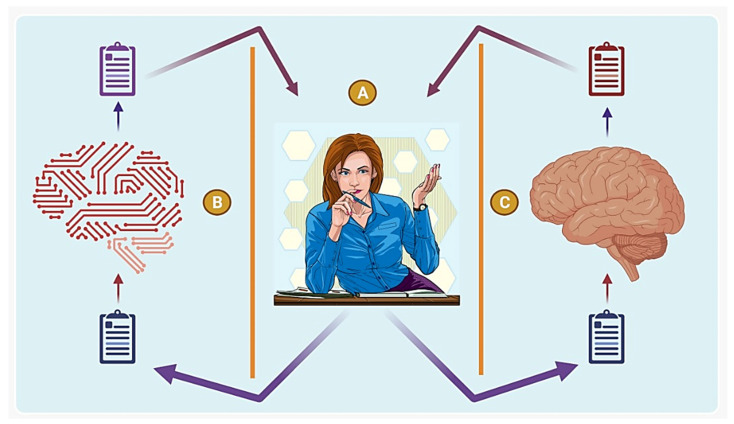
**Alan Turing designed the Turing Test in 1950.** This test includes three participants, a human interrogator, an intelligent machine and another human who we can call A, B and C, respectively. A is not aware of the identity of B and C, and A can send and receive response in only the form of text messages from B and C. A may ask B and C, a variety of questions, and based on their response, if A is unable to distinguish which one of B and C is a computer, then computer B may be considered as intelligent with thinking ability. If a human interrogator A could not distinguish the difference between another human and a computer, then the computer must be intelligent enough to be considered human. This test simply is to figure out whether or not a machine has ability to think.

**Figure 2:**
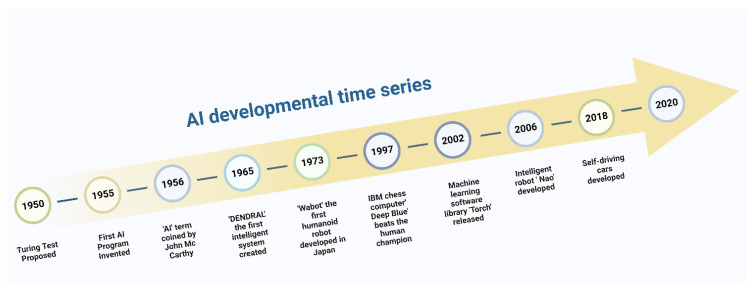
Timelines highlighting the important breakthroughs in the evolutionary path of artificial intelligence and its application in various fields.

There is no precise definition of artificial intelligence (AI) so far, but in general it refers to the ability of any machines which can simulate the intelligences of higher organisms. The field of AI has important roots in almost every branch of research including philosophy, mathematics, computing, psychology and biology [1]. An ideal AI system would be self-aware, logical and able to learn from experience. It would also be able to perceive and react to external environments. With the aid of algorithms based on machine learning (ML) and deep learning (DL) approaches, such an intelligent system could be developed to carry out activities that require human intellect [2]. John McCarthy in the 1956 first coined the term “artificial intelligence (AI) for an intelligent machine system at the Dartmouth Conference [2]. The earliest significant work in AI includes the contribution of mathematician Alan Mathison Turing. He proposed his ideas in a public lecture in London about the concept of self-learning and self-instructed machines that learn from their own experiences as a human being does [3,4]. Due to his initial observation and conceptualization of facts about smart machines, Alan Turing is widely regarded as the father of AI and modern computer science. He was an early proponent of the theory that the human brain functions essentially like a digital computer [5]. He pioneered the experiment known as “The Turing Test”, which became a pivotal moment in the development of AI (Figure 1). His paper titled ‘Computing Machinery and Intelligence,’ looked into the possibility of a non-living computer thinking like a human and was a landmark in this area [3]. Several other additional significant events paved the way for the development of the AI we see today (Figure 2). An AI program written by Arthur Samuel in 1952 for the IBM 701 prototype and a ‘virtual rat’ trained to move through a predefined path based on a neural network by John Holland were such groundbreaking preliminary works [6,7]. In 1973, a group of Japanese engineers created the first humanoid robot, which had several distinct capabilities for a machine at the time, including the ability to walk upright, hold objects and converse in Japanese.

**Figure 3:**
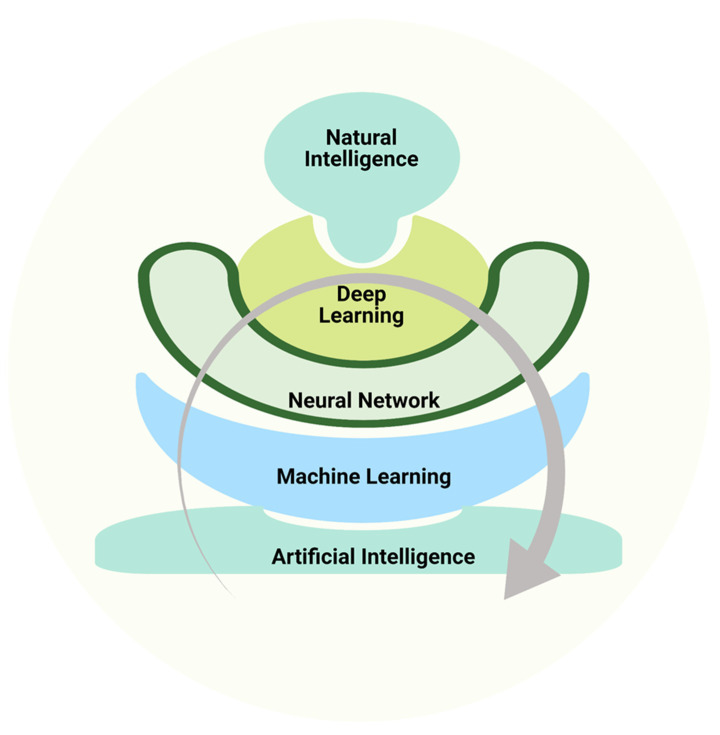
Schematic representation of major components of artificial intelligence and the continuous learning process with the help of natural intelligence to make smarter machines.

Another significant event in the AI timeline was the construction of IBM’s supercomputer, Deep Blue, which was capable of playing chess completely indistinguishable from humans. It was the first artificial intelligence to defeat Grandmaster Garry Kasparov in a timed match [8] (Figure 2). Successful use of AI planning and perception approaches may be seen in NASA’s space-based autonomous vehicles, which use technology to steer and move on their own without human intervention [4]. DL and ML are crucial elements of AI that train themselves by picking up knowledge from data of various sources that are either generated directly or indirectly from the natural intelligence system. The more these deep learning and machine learning algorithms are trained using data from various sources, the more advanced, intelligent and self-aware artificial systems may be developed (Figure 3) [9].

AI may be classified into two broad categories, Narrow or Weak AI and Artificial General Intelligence or Strong AI. Weak AI makes some attempt to copy or mimic human cognitional thought; it enables the automation of the majority of tasks in ways that humans are incapable of [10]. The most visible examples of weak AI on a daily basis include Tesla’s autopilot feature, facial recognition on our smartphones, Google’s search engine, Instagram’s AI for understanding user interests, Apple’s Siri and Amazon’s Alexa. Strong AI is a far more advanced and complex notion than weak AI. Strong AI is not restricted by human-made laws, and it thinks and controls the system entirely on its own. In layman’s terms, it is a program or a machine that simulates precise human cognitive or intellectual qualities, such as emotions or strong problem-solving abilities [11,12]. A weak AI program is designed to accomplish only one task at a time; a strong AI can efficiently perform numerous tasks simultaneously [13]. Although, self-awareness is the most essential and unique quality that distinguishes strong AI from weak AI, it is still in the early stages of development, and there are no real-life applications we can observe [13].

There are techniques used in AI which include a lot of variations, for example, the rule-based systems that are based on symbolic representations and work on inferences. AI systems have an ANN-based system which is designed to work on the interface with other neurons and connection weights [14]. Despite all of these, they all share four characteristics. Firstly, they have the feature of knowledge representation. Rule-based systems, frame-based systems and semantic networks use a series of if–then rules, whereas artificial neural networks use connections and connection weights [15]. Second, AI engineered systems are capable of learning. As self-learning entities [16], they gather data, such as by choosing the appropriate connection weights for an artificial neural network or defining the rules for a rule-based expert system. Third, they have the rules which can be implicit or explicit in an AI system. The fourth is the search, which can be incorporated into the system in several ways. For instance, it can be used to find the states that lead to a solution more quickly or to find the best set of connection weights for an ANN by minimizing the fitness function [1]. Depending on the algorithm employed, AI can also be divided into “rule-based”, also known as AI in general terms, and “non-rule-based”, also known as ML. In rule-based algorithms, conditioned branching and instructions are provided in order to obtain the best solution. For instance, the algorithm would be completely true to the instruction and merge the numbers when the case is defined as, “when subject numbers of two different datasets are the same, they should be treated as duplicates and need to be merged”. A rule-based algorithm works well when there are few options available. However, the development of a rule-based algorithm is quite challenging in complex scenarios. ML, on the other hand, develops rules directly from established training input and implements them in the ML algorithms via statistical methods. Thus, ML focuses on quickly recognizing patterns from a huge volume of information to provide findings that are more reliable than manual analysis and predictions [17].

AI has now made its way into the biological field, demonstrating its worth through innovative and cutting-edge procedures [18]. Additionally, the world has seen a true revolution in the field of information technology (IT), leading to the production and storage of an enormous amount of data, not just in the field of technology but in other areas as well in recent years. Both information technology and biology have flourished during the past half-century. According to Moore’s law, the number of transistors on a chip will double about once every two years. It is a consequence of and driver for the rapid growth of information technology [19]. Computational resources are inextricably linked to big data, which encompasses annotated and raw information due to the ever-increasing volume and complexity of data from multiple sources [20,21]. Because of developments in sequencing and other high-throughput techniques, the biosciences and biotech industries have made remarkable strides in recent years [22]. AI-based algorithms have the capacity to effectively store and process large amounts of raw, unstructured data and make them available for quick extraction, which is necessary to build an intelligent computing system with complex decision-making capability [23,24]. Such advancement in data generation, storage and analysis allows the development of a wide range of products and services in different sectors including biosciences [19]. While advances in computing and the Internet ushered in the third industrial revolution and laid the groundwork for AI’s meteoric rise, Big Data and the analytics it spawned have allowed us to take our intelligence to new heights [25]. AI is now considered a major invention of the fourth industrial revolution [26]. Experiments that would have taken years to execute are now feasible and often inexpensive due to recent advances in data and methodology. Raw data in a variety of formats are generated as a result of these experimental analyses. The ability to store and analyze data with the help of AI has created new possibilities for the academic community, scientific researchers and the biotech industry. Various applications of AI are used in biology, including the precise identification of the 3D geometry of biological molecules such as proteins which is one of the most critical tasks and useful in biological research. Moreover, in biological science, AI plays a critical role in promoting innovation not only in laboratories, but also throughout the lifecycle of a medication or chemical product [27]. Furthermore, AI-based tools and applications help automate complicated production procedures, thereby meeting the fast-rising demand for medications, chemicals for use in industry and food and other bio-based raw materials. ML, a subset of AI, aids in the prediction of outcomes by executing massive permutations and combinations of datasets available for the drug molecules to determine the best combination without relying on traditional manual methods in the lab [28]. Although traditional model-driven methods are still useful for analyzing biological data, they lack the ability to use vast amounts of available data, or even big data, to uncover information, forecast data behavior and comprehend complex data linkages. The extensive use of big data is becoming increasingly important in biotechnology and bioinformatics as it continues to grow and becomes available to academicians and scientists for analysis throughout the world [29]. These data are quantified in terms of multi-omics, such as genomics, transcriptomics, proteomics, and metabolomics, from different biological sources and need to be properly annotated and analyzed to understand complex biological systems. AI and deep neural network designs might efficiently analyze genomic data to determine the genetic basis of a trait and to uncover genetic markers linked with certain traits [30,31]. The use of AI may aid in deciphering complex links across diverse information hidden in data to obtain meaningful insights from them. As a result, the incorporation of AI approaches is now widely observed in the field of biological science and is expected to increase further in the near future as this technology matures [2]. Furthermore, medical images and drug responses contribute complex but significant data and need efficient algorithmic programs to analyze them. Therefore, ML- and DL-based AI is garnering much attention due to their capabilities for faster processing of huge data and extraction of meaningful information. AI-based digital image processing, drug designing and virtual drug tests might transform medical science in the near future [32,33]. 

The current review article highlights how Artificial Intelligence, and its components could be used in the medical, agricultural, and bio-based industrial sectors to make human life more sustainable.

## 2. AI in Medical Science

**Figure 4:**
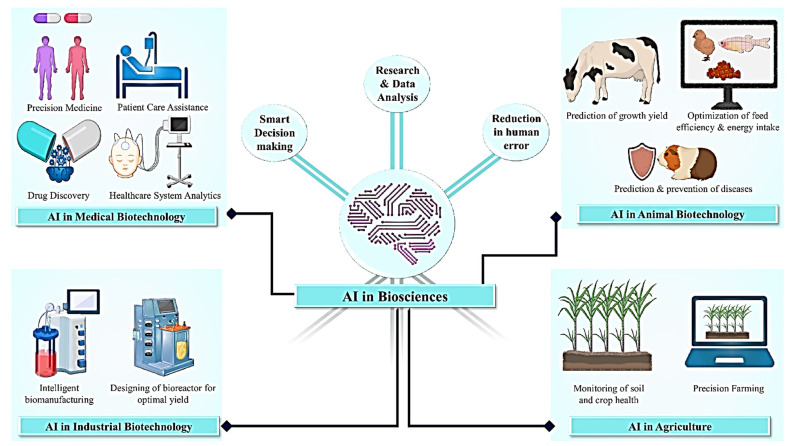
A conceptual model that illustrates the application possibilities of artificial intelligence in the disciplines of health, agriculture, animal science and industrial biotechnology.

Medical science and biotechnology advancements have opened new avenues for developing medications and antibiotics. AI has enormous potential for widespread applications in the pharmaceutical industry (Figure 4). With AI, novel therapeutic molecules based on known target structures can be discovered [34]. A branch of AI known as ML is commonly employed in disease diagnosis since it leverages the outcomes of diagnostic testing to improve the accuracy of results [35]. AI allows researchers to manage challenging issues, including quantitative and predictive epidemiology, precision-based medicines and host–pathogen interactions [36]. AI can help in disease detection and diagnosis and make computer code more accessible to non-technical individuals [37]. Predictive epidemiology, individual-based precision medicine and the analysis of host–pathogen interactions are examples of research areas that could benefit from machine and deep learning breakthroughs [38]. These approaches aid with disease diagnosis and individual case identification, more accurate forecasts and fewer mistakes, faster decision making and better risk analysis (Figure 4). The growing number of tissue biomarkers and the complexity of their evaluations significantly promote the use of AI-based techniques. These AI-based biomarkers help physicians in the prediction and analysis of the diagnosis, patient responses to the treatment and patient survival [39]. More realistic models of complex socio-biological systems are achievable because of knowledge representation and reasoning modelling [40]. ML-based methods can also be used to improve the efficiency and reliability of epidemiological models [41,42]. ML advances helped develop ten cellular parameters algorithmic program-based models that can accurately distinguish benign from malignant tumors [43]. 

It is important to take into account individual differences in genetics, ecology and lifestyle in precision medicine [44]. Medical practitioners recognize that the metabolic, physical, physiological and genetic makeup of an individual affects how their body responds to drugs in a certain way. Despite this, we are currently employing an umbrella approach that treats all patients, regardless of their varying conditions, with the same drug. However, due in large part to advances in AI, a new era of personalized medicine, in which pharmaceuticals are tailored to the body’s needs and adaptability, is evolving. Although the transition appears to be simple, it entails a significant amount of data collection, processing, maintenance and execution [45]. Moreover, millions of prediction analyses will be included in the process to identify the best therapeutic candidate molecules for a particular case. Using this strategy, physicians and clinicians may better predict which disease treatment and preventative strategies will be most effective for particular patient groups (Figure 4). Researchers could use AI in DNA, RNA and protein studies to better visualize the effects of drug doses on living tissue over time and reorganize signaling networks during therapy [46,47]. Based on AI, IBM Watson assists in the creation of the appropriate treatment plan for a patient depending on the patient’s medical history and personal data, including genetic makeup [48]. An AI-based system of personalized medicine will not only reduce treatment cost but also minimize the side effects of drugs in the patient [49]. In addition to saving time and improving patient care, AI can also simplify gene editing, radiography and drug management planning procedure [50]. Furthermore, electronic health records (EHRs) can be improved with evidence-based clinical decision support systems [44,51,52]. AI involves massive processing capacity (supercomputers), algorithms that can learn at a phenomenal rate (deep learning) and a new strategy that utilizes physicians’ cognitive talents (Table 1). This technique can contribute to the development of innovative theoretical models of disease pathophysiology and can help forecast major adverse effects of prolonged medications [53]. In a recent study, an AI-based approach was found to be very beneficial for the early identification, diagnosis, prognosis and treatment of myopia [54]. In cardiology, dermatology and oncology, deep learning algorithms outperform physicians at least in the diagnosis of disease [55,56,57]. Evidently, computer algorithms can detect metastatic breast cancer in sentinel lymph node biopsies in full slide images with an accuracy rate of more than 91 percent, and this was raised to 99.5 percent when physician inputs were added [58]. One of the proven applications of AI in risk analysis is for diagnosing heart malfunctioning through cardiovascular imaging. It includes automated monitoring of any deviations from normal conditions based on image processing, myocardial function and the detection and analysis of coronary atherosclerotic plaques [59]. The YOLOV3 algorithm was used for AI-based medical image segmentation for 3D printing and naked eye 3D visualization to detect the prostate in T2-weighted MRI images (AIMIS3D) [59]. There are several variables that might be efficiently analyzed through AI, such as determining which conditions are resistant to certain antibiotics and not to others [60]. Such analysis can support physicians and significantly decrease unnecessary testing and costs in medical care.

It is important to underline the importance of combining these algorithms with medical expertise (Table 1). New pharmaceutical compounds can be discovered via data analysis using AI, which reduces the need for clinical trials, allowing medications to be brought to market more quickly without compromising their safety [32]. Moreover, we may be able to forecast the onset of genetically predisposed diseases considerably earlier with the help of AI [50]. Patients will also be able to prevent and treat certain inherited diseases.

One of the applications of AI in the pharmaceutical industry is “Open Targets”, which is a relatively new strategic effort to explore the relationship between drug targets and diseases, as well as how certain genes are linked to diseases [67]. SPIDER is another AI technique that is being designed to determine the role of natural products in drug discovery [68]. Furthermore, quantitative structure–activity relationship (QSAR) studies are particularly useful in creating novel effective medications in a very short period of time using a computer simulation tool [69]. A QSAR model based on a radial basis function (RBF) artificial neural network (ANN) model that was trained using particle swarm optimization (PSO) technique was used in a recent study to predict the pKa values of 74 different types of drugs [69]. Natural language processing (NLP), ML, and robotic process automation are clearly the three key areas of advancement for AI in the field of medicine [70,71]. Natural Language Processing has recently been used to enhance colonoscopy analysis, improving accurate detection of adenoma and polyps [72]. Additionally, an ML approach may be used to predict diseases such as atrial fibrillation and urinary tract infections in certain patient groups by using models such as support vector machine (SVM) based on clinical features of the disease [73,74,75]. Similar initiatives have been utilized to improve heart disease prognosis using a heart-murmur-detecting technology [76]. The FDA has already approved up to 29 AI-based medical devices and algorithms in various fields of medical sciences [77].

The first AI-based model approved by the FDA in the healthcare sector was a diagnostic model based on an autonomous AI system, IDx-DR. This model was successfully used in to detect diabetic retinopathy with sensitivity, specificity and imageability of 87.2%, 90.7% and 96.1%, respectively, in a sample size of 819 subjects over 10 primary care units in the United States. The model was trained with a diversified sample dataset consisting of individuals of different ages, races and sex, thus minimizing the chances of errors in different groups [78]. Several randomized clinical trials (RCTs) have also been performed to test the efficacy and safety of AI and ML models in clinical practice. In an RCT (Registration number: ChiCTR-DDD-17012221), the impact of a deep-leaning-based automated polyp identification algorithm on polyp detection accuracy and adenoma detection rates (ADRs) was evaluated. In this RCT, successive patients were randomly assigned to go through colonoscopy either with or without the help of the automated polyp identification model that provided a simultaneous optical notification and sound alert upon polyp discovery. Results obtained from patients who have undergone the automated AI-based detection system outperformed the control cohorts of ADR and the average amount of adenoma and polyps detected per coloscopy. This automated technology can thus be pertinent in treatment regimens and routine practices for improved identification of colon polyps due to its great sensitivity, high precision and stable outcomes [79]. The introduction of AI systems in medical decision making has also resulted in the cost-effectiveness of complete medical treatment. In a study, the use of a procalcitonin-based decision algorithm (PCTDA) for hospitalized sepsis and lower respiratory tract infection patients led to a shortened duration of stay, lowered antibiotic administration, lesser artificial ventilation periods and decreased number of patients with infections and antibiotic resistance. On average, PCTDA-based treatment brought about a 49% and 23% decrease in overall expenses from conventional treatment for sepsis and lower respiratory tract infections, respectively [80]. The pharmaceutical industry will better grasp genetic information with improved AI and ML skills (Figure 4). Evidently, when integrated with ML and NLP, robotic process automation has significant applications and has the potential to reshape medical science in the near future [81]. Despite the tremendous advancements we have observed, there is still a lot of work to be done before AI-based therapy becomes a reality.

## 3. AI in Agricultural Biotechnology

Face recognition [82], cancer prediction in tissue [83] and metabolic flux analysis [84] are just a few examples of significant advances made with AI approaches, and there is a potential to achieve a similar revolution in the agricultural field. According to a report published by the United Nations’ Food and Agriculture Organization (FAO), the world’s population will reach more than 9 billion by 2050 [85]. Population expansion will eventually put a strain on the agriculture sector’s ability to provide food. In order to feed the world’s growing population and advance the nation’s economy, agriculture is essential [86]. It is a significant source of revenue for a number of countries, including India.

**Figure 5:**
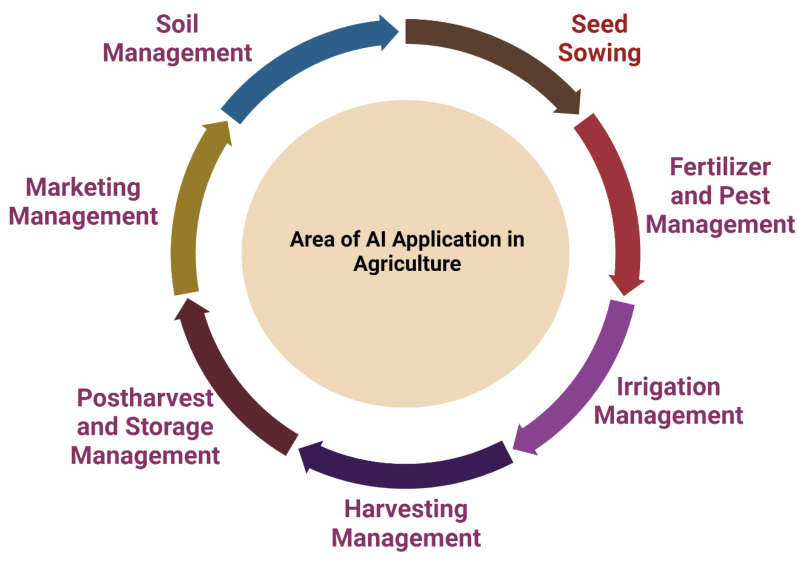
Agricultural fields where artificial intelligence (AI) could have a positive impact.

Agriculture occupies around 38% of the planet’s total land surface [85]. The majority of agricultural activities are now manual, and agriculture may significantly benefit from automation in terms of obtained yield and invested inputs. The implementation of technological breakthroughs in agriculture may contribute to the change in rural economies and villagers’ livelihoods [87,88]. Agricultural techniques are generally designed to overcome a variety of obstacles, including pest infestation, inefficient use of pesticides and fertilizers, weeds, drought and a lack of an adequate irrigation system, inefficient harvesting, storage and finally marketing. The agricultural sector could be transformed by AI intervention in the areas of soil management, water requirement assessment, precise mapping of fertilizer need, pesticide, insecticide, herbicide need, yield prediction and overall crop management (Figure 5) [89,90,91]. With the advancement of AI-based technology, drones and robots are being used to improve real-time monitoring of crops, harvesting and subsequent processing [92]. AI and ML techniques are currently being used by biotechnology companies to design and train autonomous robots capable of performing key agricultural activities such as crop harvesting at a much faster rate than traditional methods [89]. The data collected by drones are processed and evaluated using deep learning and computer vision techniques [93]. Machine learning approaches assist in the access and forecast of a wide range of environmental variables that influence agricultural output, such as weather fluctuations and the arrival of the monsoon in India [89,94,95]. As mentioned elsewhere, AI-based solutions in the agricultural industry help to improve efficiency and control numerous aspects such as crop yield, soil profile, crop irrigation, content sensing, weeding and crop monitoring (Figure 5) [89,96].

Traditional and older morphological characteristic inspection is time-consuming, error-prone and costly. The machine vision method might be easily applied in agricultural practices, which can speed up and simplify the procedure while being more precise and accurate [93]. Identification and selection of improved varieties may speed up and make the process easier by using automated non-invasive, rapid scoring of various plant features through high-throughput phenotyping methods [97]. Due to the tools of AI and IoT, swarm intelligence and drone technology can now be employed for several agricultural activities [98]. Recent developments in DL- and ML-based algorithm design to estimate the price of agricultural products may enable farmers to receive a higher return on their labor and investment [99]. For effective irrigation, artificial neural networks, fuzzy logic and meta-heuristic algorithms have recently been developed [100,101]. According to a recent study, convolutional neural network (CNN), which takes into account several environmental variables, is one of the most trustworthy ML algorithms to estimate soybean and maize yields [102]. Recent advances in AI-based biosensors for early disease detection in crop plants, even in asymptomatic plants, have the potential to greatly minimize product loss caused by biotic stressors [103]. AI-based drone technologies such as EfficientNetV2, which are designed to detect and classify plant diseases with accuracy and precision of 99.99% and 99.63%, respectively, are one of the promising automated technologies for the monitoring of plant health in a time-saving and cost-effective manner [104]. For the detection of bacterial spot disease in plants, a hybrid AI model based on convolutional autoencoder (CAE) and CNN has also achieved 99.35% and 99.38% in the training and testing periods, respectively, [105].

The use of AI may make it simpler to identify potential targets in big genome data for genetic manipulation and design effective synthetic promoters in efforts to improve agronomic traits in plants [106,107]. The growing necessities for smart agriculture have resulted in substantial advancements in the area of AI-based agricultural forecasting and prediction, which has improved crop productivity to a great extent [93]. A similar attempt was made in a recent study where image datasets were analyzed by employing AI algorithms, namely ANN and genetic algorithm (GA)-based platforms, for the prediction of crop yield in an optimized manner [108]. During the training period, the model obtained a maximum validation accuracy of 98.19%, whereas a maximum accuracy of 97.75% was yielded during the test period [108]. This model worked effectively under limited resource restrictions and less data, producing optimal results [108]. In another significant study, a new methodology for predicting agricultural yield in greenhouse crops employing recurrent neural network (RNN) and temporal convolutional network (TCN) algorithms was proposed [109]. Based on previous environmental and production data, this approach can be utilized to estimate greenhouse crop yields more accurately than its standard ML and deep learning peers [109].

Furthermore, this experimental investigation has also demonstrated the crucial importance of previous yield datasets in correctly predicting future crop productivity [109,110]. Several million individuals in developing nations have benefited from the green revolution by preventing and combining high-yield crops, synthetic fertilizers and water. However, owing to widespread misuse of herbicides, pesticides and fertilizers, the green revolution could not be considered fully “green”. Certain approaches for high-yielding crops typically need a large amount of agro-chemicals and water [111]. AI-based approaches are being developed to reduce the reliance on noxious agro-chemicals and to attain a state of sustainability in agriculture [79]. For optimizing agricultural resources, a remote sensing assisted control system (RSCS) has been developed [112]. This methodology makes use of AL and ML technology to improve environmental sustainability while fostering novel agricultural product development planning. When analyzed with other techniques, the findings revealed that the RSCS demonstrated the highest precision, performance, data transfer rate, productivity, irrigation management and carbon dioxide release ratio of 95.1, 96.35, 92.3, 94.2, 94.7 and 21.5%, respectively, [112]. Thus, AI models have the potential to manage agricultural products and productivity in a “green” manner. In another study, an AI and machine vision-based smart sprayer was developed to spray herbicides specifically to weed targets, thus reducing weedicide overuse and environmental contamination. This sophisticated technology combined a cutting-edge weed detection concept, a unique rapid and precise spraying method and a weed mapping model with 71% and 78% precision and recall, respectively, [113]. Due to limited collecting techniques and a lack of integration of diverse data sources, data gathering from agricultural regions linked to soil hydration, crop quality or insect infestations frequently depend on manual analysis.

Meanwhile, as the industry becomes more digital, the combination of remote sensing for computerized screening and analytical techniques with datasets for soil studies, weather predictions, etc., and sophisticated AI models is reducing the need for agrochemicals [93]. In this regard, the substantial NaLamKI action plan that seeks to create AI-based open access software that could greatly help the agricultural industry has received funding from the German government. This plan seeks to develop datasets by combining information from different sensors in order to optimize different farming practices with the help of AI and ML technologies [93,114]. Similar governmental initiatives are required in large numbers to make farmers adapt AI on a greater scale.

In agriculture, integrating precise image-based features with omics data may aid in finding critical traits involved in stress tolerance and acclimatization mechanisms [115], as well as contribute to the development of climate resilient cropss. Farmers will be able to generate more output with less input, increase the quality of their output and ensure a faster time to market for their harvested crops owing to AI-based technology adaptation [93] (Table 2). Although first-generation AI can be employed in the surveyance and classification of omics data, it is tailored for the handling of specific problems related to single-omics datasets without integrating data from other modalities [93,116]. In agricultural biotechnology, next-generation AI is fundamentally envisioned to dynamically ameliorate and handle large multi-omics datasets in addition to predicting the breeding value of complex traits across different environmental conditions [116].

## 4. AI and Industrial Biotechnology

Industrial biotechnology, sometimes known as white biotechnology, is the modern application of biotechnology to the sustainable processing and manufacturing of commodities, chemicals and fuels from renewable sources using live cells and their enzymes. The demand for industrial chemicals, medicines, food-grade chemicals and other biochemistry-related raw materials has increased dramatically over the previous decade [121]. ML and AI-based technologies may aid in the design of novel pharmaceuticals and the identification of their efficacy and adverse effects before their actual production, drastically reducing the time spent bringing a drug from the lab to the market for ordinary people [32]. Microorganisms and plant/animal cells are used in biotechnological processing to make products in a variety of sectors, including drugs, pharmaceuticals, food and feed, disinfectants, pulp and textiles. In order to detect outages, optimize machinery for efficient manufacture and improve product quality, the Internet of things, ML and AI could be used effectively [122]. AI-based computer models are becoming increasingly widespread, and robotics and machine learning could be used to develop the best optimum growth conditions for the strains, as well as the degree to which valuable products can be obtained (Figure 4). For instance, AI or response surface methodologies (RSM) -based approaches have been used in the high-level production of amylases from *Rhizopus microsporous,* using various agro-industrial wastes for optimal experimentation designs [123]. Similarly, AI algorithms such as artificial neural networks (ANN) and genetic algorithms (GA) have been integrated for the optimization of fermentation media to produce glucansucrase from *Leuconostoc dextranicum*. A 6% rise in glucansucrase activity was predicted by the integrated ANN-GA model over a regression-based prediction approach [124]. The application of the integrated ANN-GA model for the optimization of cellulase production by *Trichoderma stromaticum* under solid-state fermentation has been reported recently, and a 31.58-fold increase in cellulase production was achieved after optimization with the AI model [125].

AI-based technologies have also been used to scale up and optimize bioprocesses for enzyme production on pilot scales. A low-cost method for increasing the synthesis of extracellular laccase from *Staphylococcus arlettae* utilizing tea waste was performed in a study. RSM and ANN coupled with GA were two consecutive statistical methods that were employed to increase enzyme production and resulted in a sixteen times rise in enzyme yield. Moreover, a pilot scale bioprocess was established utilizing the ideal parameters identified by GA, namely tea waste (2.5%) NaCl (4.95 mM), L-DOPA (5.65 mM) and 37℃ temperature, which improved the enzyme production by 72 times [126]. Furthermore, some AI models based on the fuzzy expert system are also capable of monitoring wastewater treatment plants on a pilot scale [127].

Biofuel is one of the most important bioproducts for which the industrial production process can be enhanced using ML and AI for maximum output. In the bioenergy sector, AI-based approaches have been used to predict biomass feedstock properties, bioenergy end-uses, and bioenergy supply chains and have developed an integrated ANN-Taguchi method model for the prediction and maximization of biofuel production via torrefaction and pyrolysis [128,129]. Optimization and design of experimental factors were performed using the Taguchi method which led to the attainment of maximum biofuel yield up to 99.42%, whereas ANN showed linear regression prediction of 0.9999 for biochar and 0.9998 for bio-oils.

Integrated ANN-GA models have been used in the modeling and optimization of the methanolysis process of waste peanut shells for the generation of biofuels. Biofuel yield optimized by the RSM model was 16.49%, whereas that of the ANN-GA model was reported to be 17.61%. This shows that integrated ANN-GA has better optimization potential than the RSM model alone [130]. ML-based bioprocess models have also been constructed with the help of AI-based methods such as ANN, CNN, (long short-term memory networks) LSTMs, kNNs (k-nearest neighbors) and RF (random forests) for predicting the accumulation of carbohydrates in cyanobacteria biomass cultivated in wastewater for biofuel production. The finest results for approximation of system dynamics were achieved with a 1D-CNN with a mean square error of 0.0028 [131]. Textiles, new chemicals and biodegradable biopolymer synthesis could all benefit from similar processes [132]. Furthermore, it may be used to assist in the development of synthesis techniques for such biochemicals that produce the highest yield with the least amount of input (Figure 4). Additionally, AI could assist in real-time forecasting of market demand for medications or chemicals. AI and ML have also helped in the production of metabolites. Systems metabolic engineering is a process that helps in the rapid production of high-performing microbial strains for the long-term production of chemicals and minerals. The increasing availability of bio big data, such as omics data, has resulted in an application for ML techniques across various stages of systems metabolic engineering, such as host strain selection, metabolic pathway reconstruction, metabolic flux optimization and fermentation [19]. Various machine learning algorithms, including deep learning, have facilitated in optimizing the bioprocess parameters and exploring a larger metabolic space that is linked to the biosynthesis of a target bioproduct [133]. This trend is also influencing biotechnology businesses to adopt ML techniques more frequently in the creation of their production systems and platform technologies [134]. In the brewery industry, AI has demonstrated promising potential to overcome fundamental shortcomings and enhance production through knowledge accumulation and automated control. In a study, AI models were constructed using aroma profiles and spectroscopic data obtained from commercial alcohol for assessing the quality traits and aroma of beer. The intelligent models resulted in highly accurate predictions for six major beer aromas [135]. Smart e-nose technologies based on ANN models have also been developed to assess the presence of different chemicals such as ethanol, methane, carbon monoxide, hydrogen sulfide, ammonia, and so forth in beer [136]. A study was involved in the development of a computer program that simulated the operation of a highly customizable three-layer feed-forward multilayer perception neural network, which using data from prior experiments, could forecast changes in the parameters of white wine alcoholic fermentation. This work provided a befitting approach for the digitalization of brewing processes, thus enabling it to be acclimatized to other intelligent and knowledge-based frameworks [137]. Another study led to the development of an innovative knowledge-based approach for controlling the batch fermentation of alcohol employed in making white wine. The primary sources of information used in developing the AI model were different case studies and experimental results, as well as the knowledge obtained from brewery experts regarding different parameters related to optimization and control of the overall process. Using the monitoring, regulation and data acquisition software of the fermentation bioreactor, an application for automated process control was developed [138]. The further incorporation of control systems, processes and innovative advancements can be greatly facilitated by such kinds of AI models, thus supporting sustainable development.

## 5. Challenges and limitations

Despite their immense potential, AI-based technologies have yet to make their way into everyday practice. AI models can improve the accessibility of various biological sectors; however, they may also exacerbate pre-existing discrepancies. Since AI models are extremely reliant on the datasets on which they are developed as well as the labels connected with them, prejudices against the underrepresented in the learning algorithms might be reinforced [139]. Several factors must be considered to properly assess the resilience of some deep neural networks. For the development of AI models, metadata must be created, retrieved and cleansed. Programs should further be designed and evaluated under the oversight of field professionals for analysis and correction of inaccuracies committed in practice [140]. In spite of significant advances in the design of AI and ML-based models in recent years, few have been incorporated into healthcare, and many prospects for adopting these models for everyday usage remain untapped. CNNs, for instance, were initially used in study designs commencing in 2015, primarily on dental radiographs, with the first clinical uses for these tools only recently emerging [141]. Unavailability and inaccessibility of clinical data due to organizational policies, insufficient reproducibility in processing datasets and assessing outcomes and residual concerns around accountability and transparency to patients remain the most common hurdles in adapting AI in routine medical and dental practices [142]. Moreover, several models have been reported to be inaccurate in predicting the clinical diagnosis. For example, an AI algorithm that can diagnose and classify chest X-rays using NLP to radiological records was developed [82,143]. These classifications were subsequently utilized in the training of a deep learning network to detect abnormalities in pictures, with a specific focus on recognizing a pneumothorax [144]. However, after a thorough examination, the presence of a chest tube in the majority of the reports identified as pneumothorax raised questions that the algorithm has been recognizing chest tubes instead of pneumothorax as envisioned [143]. Another example of non-interpretative results of a clinical AI-based system is DeepGestalt, a tool for analyzing facial dysmorphology. This tool performed poorly when identifying people with Down syndrome who were of African heritage (36.8%) compared to those who were of European origin (80%) [145]. The diagnoses of Down syndrome among people of African lineage increased to 94.7% when the model was retrained using cases of people with the condition [145]. Due to various marginalization in training datasets, genetic disease susceptibility modeling is also predisposed to differential performance across demographic groups [146]. Furthermore, it has been observed that while ML approaches may perform better in studies for developing disease risk prediction models, the presentation of the data may be more complex. There is also a possibility that the amount of computational time required by ML approaches varies depending on the size of the data [147]. Thus, it is crucial to acknowledge that the utilization of AI-based approaches will not always lead to improvised categorization or better prediction than present methods. AI is a tool that should be employed within the proper context to address a pertinent question or resolve a significant issue [148]. Similarly, in other biological fields such as agriculture, automation in practices employing AI and ML-based approaches leverages a lot of potential for sustainable farming. However, in the agricultural sector, the collection, analysis and utilization of data for productivity present a number of obstacles. Privacy and security of data are the two major challenges that farmers must address to survive in the digital age. In most cases, the farmers are uninformed of the collection, usage, and more concerningly, the purposes for which their personal details are being utilized [149]. Data mining allows corporations to rely on individuals in order to acquire massive agricultural data, which may be sufficient to develop and evaluate the behavioral and psychiatric pictures of the respondents [150]. To stop data from being misused, farmers require assurance that their information will be utilized to generate innovative ideas and agricultural solutions rather than to gain a competitive advantage. As mentioned elsewhere, the AI-based drone technology has emerged as a highly effective approach in agriculture [87]. However, drones, particularly those equipped with high-resolution lenses, infrared cameras, competent programs and sensors, are highly expensive for small farmers. Moreover, to operate drones, one needs authorization according to its operative and regulative provisions of the law of land [151]. Furthermore, weather imparts a huge influence on the operation of drones [152]. Traditional data mining methodologies are primarily developed for relational datasets; however, they are not completely adequate for geographically scattered data [153]. To revolutionize agriculture with AI-based technologies, innovative data mining approaches are needed.

In the industrial biotechnology sector, establishing defined and viable protocols for adopting an algorithm and assessing dataset size remain a major challenge. To design such protocols, it would be necessary to have a thorough knowledge of the effects/efficacy of various algorithms as well as training datasets to address numerous bioindustry challenges. Furthermore, increased accessibility, good documentation and superior data acquisition methods are still required to develop, operate and optimize bioenergy systems and bioreactor designs [128]. In some AI models, when the input is inadequate, particularly for large dimensional datasets, the algorithm may only recall every single variable as a special instance instead of learning the information, resulting in errors and lower training efficacy [154]. Additionally, numerous ANN-represented systems are frequently chastised for having black-box characteristics. Nonetheless, the paucity of comparative works across different AI–ML designs renders it challenging to present a clear direction for future studies or practical implementation [155]. There still exist challenges that need to be overcome including inefficient data integration which arises due to the diversity of the datasets inclusive of candidate data, metadata, processed data, raw data and lack of proper skill set and expertise related to the subject [156]. In this context, it is necessary to overcome these ambiguities by utilizing new AI algorithms to achieve a thorough alignment between the anticipated outcomes and the empirical studies [157]. Thus, more extensive datasets and relative studies are required to develop AI and ML-based models for real-time monitoring and control of bioreactors and bioprocesses.

## 6. Conclusions

One of the great achievements we have seen in the era of Industry 4.0 is the ability of a machine to replicate the capacities of living systems, particularly the intelligence of a human. The ability to recognize objects and make decisions is a crucial characteristic of biological systems. AI can currently recognize objects and make decisions using many of the cognitive and perceptual abilities of live systems. The potential of AI might be utilized to the biological world, including medical research, agriculture, and bio-based industries, for our sustainable way of life. The early prediction and identification of disease and its precise treatment based on personalized medicine even while the diseases are in asymptomatic conditions are examples of key areas in medical science that might benefit from AI. This would not only save millions of lives but also reduce medical costs. In addition to the medical field, AI-based efficient algorithms and programs have been recently developed to ensure effective inputs and outputs in farming, a practice known as precision farming. Agricultural practices such as soil management, water need analysis, exact modeling of fertilizer requirement, pesticides, insecticides, herbicides, yield projection and overall crop management could also be revolutionized by AI intervention. This would help to meet the world’s rising population’s demand for food. When we talk about large-scale production, many variable factors lead to increasing costs, which are major challenges. Recently, AI-based programs and computer models have proven to be very efficient at optimizing the suitable conditions to obtain the maximum desired product, whether for agricultural, medical, biotech, or lifestyle uses, at minimum cost. The efficient production of bio-enzymes is just one of such successes, and it is easy to envision how the biotech industry will be transformed by the application of AI, which will help to reduce production costs, one of the biggest challenges facing the industry today.

## Figures and Tables

**Table 1:** AI in disease detection and prediction modelling.

Diseases Studied	Algorithm	Modality	Findings	References
AMD	ML-based predictive model	Clinical data	AI-based predictive model was able to predict the progression of AMD with high accuracy	[61]
Alzheimer’s disease	RF, SHAP	Clinical and Imaging data	AI-model was able to accurately detect and predict the progression of Alzheimer’s disease with accuracy of 93.95% in first layer and 87.08% in second layer	[62]
COVID-19	PA	Clinical data	An accuracy of 70–80% was achieved inn predicting severe COVID-19 cases	[63]
Ovarian cancer	ANN	Clinical data	An accuracy of 93% was achieved in predicting the survival of ovarian cancer patients, and 77% accuracy was achieved in predicting the surgical outcome	[64]
Pulmonary cancer	LCP-CNN, Brock model	Clinical data	LCP-CNN was able to predict the malignancy of pulmonary nodules with higher accuracy and lower false negative results than Brock model	[65]
Influenza	IAT-BPNN	CDC data and Twitter dataset	IAT-BPNN was able to predict influenza-like illness in a large population size with an high accuracy	[66]

**Table 2:** Recently developed AI-based algorithms in the agricultural sector.

Aim	Algorithm	Sample Size	Results	References
Salmonella occurrence and absence prediction in agriculture streams	ANN, kNN, SVM	400	Tested algorithms predicted Salmonella presence with an accuracy ranging 58.15–59.23%	[117]
Prediction of *Oryza sativa* L. growth rate modelling	REG, ANN, GEP	95	Simulation of growth rate was predicted better with ANN & GEP than REG	[118]
Detection of seed germination	CNN	16	An average of 97% seed recognition accuracy was achieved	[119]
Detection of tomato and mass estimation	Mask-RCNN, ResNet101-FPN, RPN	-	A detection accuracy of 99.02% with a precision of 99.7% was achieved	[120]
Designing of smart tree crop sprayer	LiDAR, machine vision, GPS, CNN	-	An accuracy of 84% was achieved in the classification of different trees; a 28% reduction rate was achieved in spraying of chemicals as compared to conventional techniques.	[113]

## Data Availability

Not applicable.
